# Validation of a novel method for canine eruption assessment in unilateral cleft lip and palate patients

**DOI:** 10.1002/cre2.397

**Published:** 2021-01-15

**Authors:** Khalid Alqahtani, Eman Shaheen, Sohaib Shujaat, Mostafa EzEldeen, Titiaan Dormaar, Maria Cadenas de Llano‐Pérula, Constantinus Politis, Reinhilde Jacobs

**Affiliations:** ^1^ OMFS IMPATH Research Group, Department of Imaging & Pathology, Faculty of Medicine, KU Leuven & Oral and Maxillofacial Surgery University Hospitals Leuven Leuven Belgium; ^2^ Department of Oral and Maxillofacial Surgery and Diagnostic Sciences, College of Dentistry Sattam Bin Abdulaziz University Al‐Kharj Saudi Arabia; ^3^ Department of Oral Health Sciences‐Orthodontics, KU Leuven and Dentistry University Hospitals Leuven Leuven Belgium; ^4^ Department of Dental Medicine Karolinska Institutet Stockholm Sweden

**Keywords:** 3‐D imaging, cleft lip, cleft palate, cone‐beam computed tomography, tooth eruption

## Abstract

**Objective:**

The aim of this study was to propose and validate a three‐dimensional (3D) methodology for the assessment of canine eruption in patients born with unilateral cleft lip and palate (UCLP) following secondary alveolar bone graft (SABG).

**Methods and Materials:**

A total of 10 patients (four females, six males; mean age: 8.8 years) with UCLP who underwent SABG were recruited. Pre‐ and 6‐month post‐operative cone‐beam computed tomography (CBCT) was acquired for all patients. Post‐operative data was registered onto pre‐operative data utilizing voxel‐based registration. Following superimposition, a segmentation process was applied to segment maxillary canine on both cleft and non‐cleft side. Thereafter, translational and rotational changes in canine position were assessed for both cleft and non‐cleft side by two observers.

**Results:**

The intra‐class correlation coefficient (ICC) indicated excellent reliability (≥0.90) with inter and intra‐observer error of less than 0.05 mm. The overall ICC was found to be high for assessing both translational and rotational changes. The mean absolute inter‐ and intra‐observer difference for translational and rotational changes was found to be less than 1 mm and 3°.

**Conclusion:**

The present method was found to be reliable proving to be clinically applicable for assessing maxillary canine eruption changes in both cleft and non‐cleft bone.

## 1 INTRODUCTION

Unilateral cleft lip and palate (UCLP) is considered to be among the most common of all craniofacial anomalies, having a worldwide prevalence of around 0.5–3 in 1000 newborns. It occurs more frequently on the left side with predominance in males compared to females (2:1) (Murray, 2002). Patients born with UCLP suffer from multiple aesthetic and functional impairments which can negatively influence their self‐confidence, communication abilities and behavior (Turner et al., 1998).

Numerous surgical techniques have been developed for improving functional/aesthetic outcomes and restoring facial symmetry in patients born with UCLP. Among these techniques, secondary alveolar bone grafting (SABG) has become an integral component for the multidisciplinary surgical management of patients with cleft lip and palate (Feichtinger et al., 2007). In addition to stabilization of the maxillary segment and filling of the cleft area, an effective SABG provides several benefits such as alar base support, oro‐antral fistulae closure and induction of teeth eruption by providing sufficient bone for further orthodontic treatment and potential implant placement in case of missing teeth (Bergland et al., 1986). In patients born with UCLP, SABG plays a vital role for facilitating canine eruption in the cleft region. An abnormal eruption of canine can lead to root resorption and damage of adjacent teeth, thereby complicating the orthodontic treatment. Therefore, early prediction of canine movement and eruption following SABG is crucial to avoid these complications (Enemark et al., 1987; Russell & McLeod, 2008).

Previous studies assessing the eruption of maxillary canines relied on traditional two‐dimensional (2D) intraoral, occlusal, periapical and panoramic radiographs (Long et al., 1996). These 2D imaging modalities are associated with certain inherent limitations, such as image magnification and distortion, structure superimposition and reduced image quality which can directly influence the accuracy of evaluating the bone graft and teeth eruption (Russell & McLeod, 2008). To overcome these limitations, cone‐beam computed tomography (CBCT) have replaced its 2D counterparts in cleft patients, which provides a more accurate assessment of the alveolar bone grafting and its relationship with neighboring teeth (Hamada et al., 2005). However, evidence suggests only a few studies assessing the 3D maxillary canine eruption in patients born with CLP (Vellone et al., 2017; Walker et al., 2005). The assessment methodologies utilized in these studies relied either on landmark identification or cephalometric analysis which is prone to human error (Katkar et al., 2013). To reduce this error, various registration methods have been proposed, which include landmark‐based, surface‐based and voxel‐based registration for superimposing similar anatomical structures and assessing the 3D changes (Hajeer et al., 2005; Shamir et al., 2009). Out of these methods, voxel‐based registration has been accepted as the most reliable method (Lee et al., 2012; Shahen et al., 2018). However, no studies have assessed canine eruption utilizing voxel‐based registration and objectively measuring how a canine translates and rotates in a three‐dimensional space in cleft patients which cannot be appreciated with the 2D methodologies.

Therefore, the aim of this study was to develop and validate a novel registration‐based methodology to assess canine eruption in patients born with UCLP.

## 2 MATERIAL AND METHODS

This study was conducted in compliance with the World Medical Association Declaration of Helsinki on medical research. Following an ethical approval from the Local Ethical Committee (reference number: S58298), a sample of 10 patients born with UCLP (6 males, 4 females; mean age: 8.8 years) who underwent SABG at the Department of Oral and Maxillofacial Surgery, University Hospitals Leuven, Leuven, Belgium was recruited. The sample size was selected based on similar validation studies with 3D assessment methodologies (Bianchi et al., 2010; Marchetti et al., 2011; Nadjmi et al., 2010). A SABG procedure was performed in all patients by harvesting an autogenous corticocancellous bone graft from the anterior iliac crest (Dasari et al., 2018).

Pre‐ and 6‐months post‐operative CBCT scans used in the present validation study have been acquired in accordance with the ethically approved and justified radiographic cleft protocol at the Leuven University Hospitals Cleft Center as described by De Mulder et al. (2018) for all the patients. The mean age of patients at the preoperative scan time was 8.8 ± 0.6 years and 9.4 ± 0.6 years post‐operatively. The scans were carried out with three acquisition devices as mentioned in Table 1 and each patient was scanned by the same machine at pre‐ and post‐operative time points. The inclusion criteria involved non‐syndromic UCLP, presence of bilaterally unerupted maxillary canine, absence of deciduous canine in cleft side before SABG and good quality pre‐ and 6 months post‐operative CBCT scans with a large field of view including the maxillary bone with infraorbital foramen. The exclusion criteria included syndromic UCLP, bilateral CLP, history of facial trauma and presence of motion artifacts.

**TABLE 1:** Cone‐beam computed tomography acquisition devices and methods

System	3D Accuitomo170	Newtom VGi‐evo	Promax 3D Max
**System origin**	J. Morita, Kyoto, Japan	Newtom, Verona, Italy	Planmeca, Helsinki, Finland
**Total mAs**	5	5	5
**Potential (kV)**	96	110	96
**Slice thickness (mm)**	0.15	0.2	0.2
**Field of view (mm)**	8 × 8	10 × 8	8 × 8

### 2.1 Eruption assessment methodology

The proposed method was implemented on both cleft and non‐cleft side between pre‐ and 6 months post‐operative time points to illustrate the clinical validity of the newly proposed method. The steps and details of the methodology are described in the following subsections.

#### 2.1.1 Registration

**FIGURE 1:**
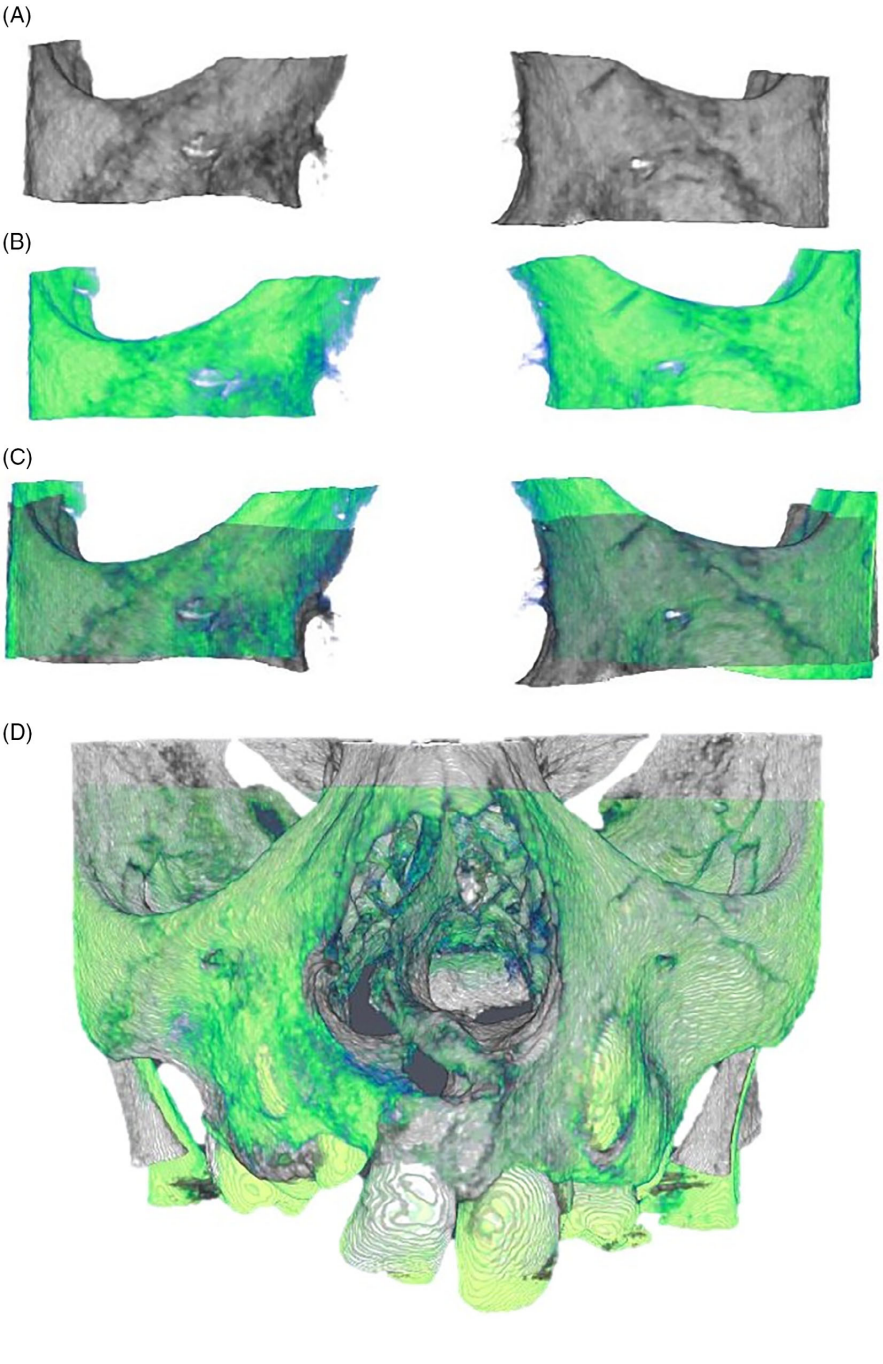
Voxel based registration procedure. (a) Preoperative region of interest for registration (gray), (b) 6 months postoperative region of interest for registration (green), (c) 6 months postoperative registered on preoperative based on the region of interest, (d) result the registration showing the full scans

A semi‐automated step‐wise tool developed in Amira software™ (edition 6.3.0, Thermo Fischer Scientific, Merignac, France) was utilized to register the post‐operative onto the preoperative scan. The registration was voxel‐based with maximization of mutual information that optimally aligns two sets of images (Maes et al., 1997). The region for registration was chosen to include the maximum amount of bony region which was unaltered during the operation and to exclude the unstable dentate region. The registration region involved the maxillary bone cropped inferiorly above the apex of the teeth roots, superiorly at the infraorbital rim and bilaterally at the zygomatic process as shown in Figure 1a–c. Figure 1d illustrates the final achieved superimposition of the pre‐ and post‐operative CBCT scans based on the defined region of interest. The pre‐ and registered post‐operative images were exported in Digital imaging and communications in medicine (DICOM) format for further processing.

#### 2.1.2 Segmentation

**FIGURE 2:**
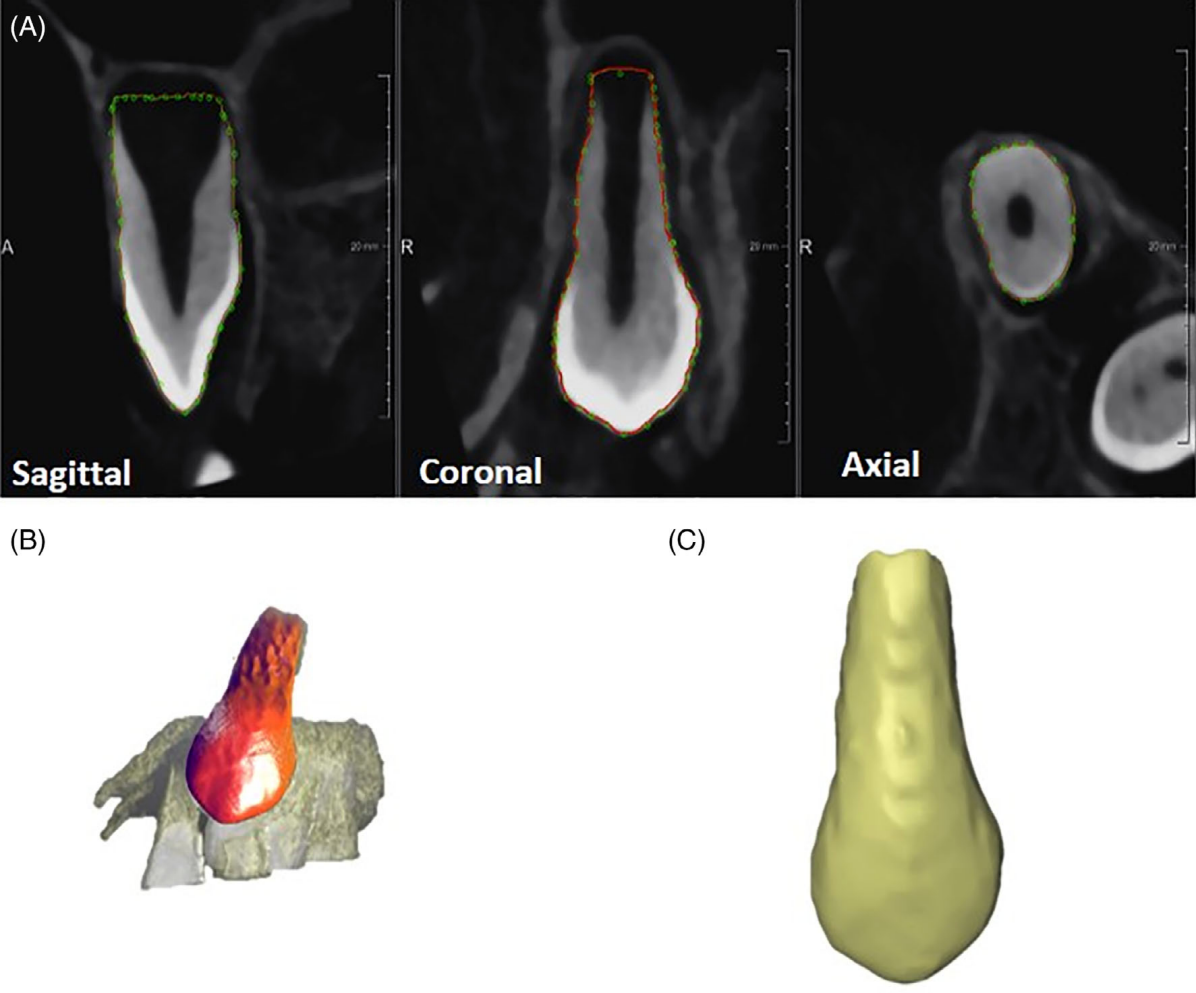
Segmentation procedure. (a) The semi‐interactive livewire step delineating canine in sagittal, coronal, and axial views; (b) the rendering of the segmented canine shown within the surrounding tissues. (c) The resulted segmented 3D model as STL

A segmentation process was utilized to extract canines on cleft and non‐cleft side from both pre‐ and registered post‐operative DICOM images. The segmentation was performed using a dedicated and validated framework developed in MeVisLab (MeVis Research, Bremen, Germany) (EzEldeen et al., 2015). Firstly, the DICOM images were imported into the framework. Thereafter, the user interactively indicated the boundaries of each canine individually by means of a livewire boundary extraction algorithm. The livewire function guides the user by automatically detecting the edges of the selected tooth and creates a contour. The contours were then extracted from the axial, coronal and sagittal views (Figure 2a). These contours were automatically combined and the surfaces of the canines were reconstructed by means of an interpolation algorithm (Heckel et al., 2011) as shown in Figure 2b. The 3D surfaces of the bilateral canines in both scans were then exported as stereolithography (STL) files. An example of the segmentation is shown in Figure 2.

#### 2.1.3 3D analysis

**FIGURE 3:**
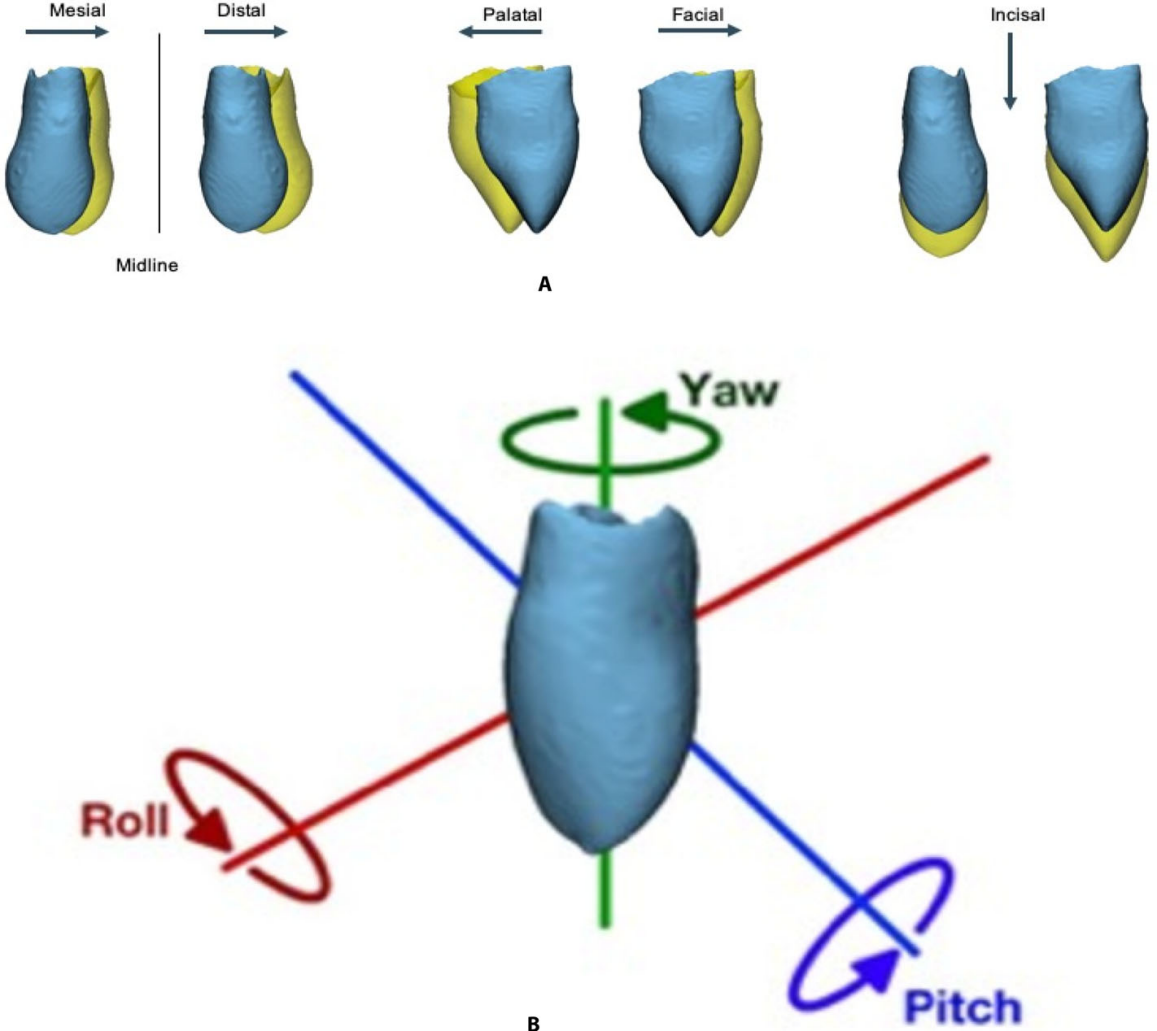
3D canine positional changes. Blue: Pre‐operative. Yellow: Post‐operative. (a) Translational mesial/distal, palatal/facial and incisal movement. (b) Rotational pitch, roll and yaw movement

The STLs of the segmented canines were imported into a fully automated module developed in Amira software. The module applied surface‐based registration between the two STL files of the canines to retrieve the transformation matrix which indicates the displacement between the canines. The transformation matrix was further decomposed using singular value decomposition (SVD) algorithm to calculate the translational and rotational changes between the pre‐ and registered post‐operative position of the canines in both cleft and non‐cleft side (Shaheen et al., 2019). The translational changes included: mesial/distal (M/D), palatal/facial (P/F) and incisal/apical (I/A) and the rotational changes were categorized into pitch, roll, and yaw (Figure 3).

**FIGURE 4:**
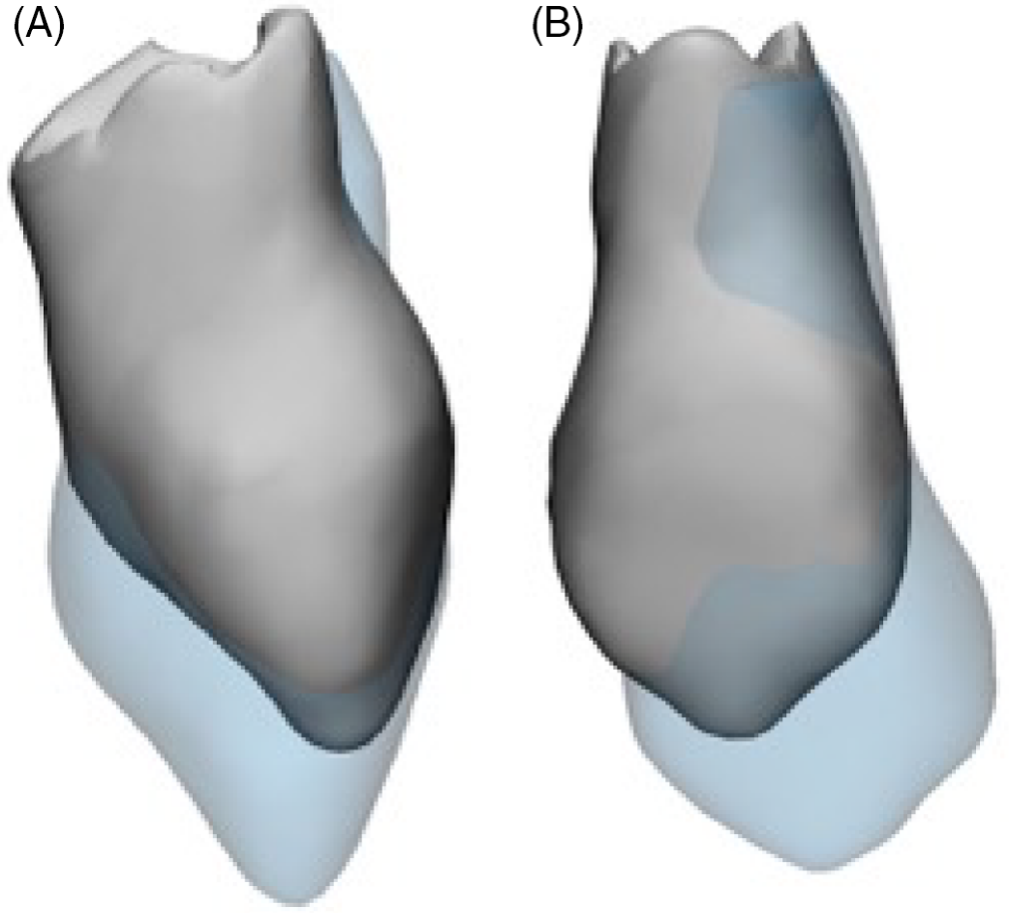
An example of a non‐cleft side erupted canine. Preoperative canine is in gray and registered postoperative canine is in transparent blue. (a) Side view. (b) Frontal view

Figure 4 shows an example of an erupted canine on non‐cleft side where the translational changes were 1.4 mm mesially, 1.4 mm facially, and 2.4 mm incisally. The rotational movements were 4.5°, 6.3°, and 6° for pitch, roll and yaw respectively.

The accuracy of the registration step involving superimposition of the pre‐ and post‐operative scans was further assessed by means of a color‐coded distance map. These pre‐ and registered post‐operative regions of interests were segmented via thresholding in Mimics software (Materialise, Leuven, Belgium) then analyzed in 3‐matic software (Materialise) using part comparison function. This allowed the calculation of the mean absolute difference (MAD) between the surfaces, where MAD indicates the error of registration between the pre‐ and registered post‐operative scans. The complete methodology and registration step was performed twice independently by two observers following training and calibration for calculating the intra‐ and inter‐observer reliability at an interval of 1 week.

### 2.2 Statistical analysis

Data were analyzed using MedCalc statistical software (version 12.0, Ostend, Belgium). Intra‐Class Correlation Coefficient (ICC) was applied at a 95% confidence interval for assessing the inter‐ and intra‐observer reliability where an ICC starting from 0.90 was considered excellent (Bartlett & Frost, 2008). The mean, absolute mean and SD were also calculated for all the data. The Wilcoxon signed rank test was applied for assessing the translational and rotational difference between cleft and non‐cleft canines.

## 3 RESULTS

Table 2 represents the ICC for the registration step at a 95% confidence interval. The ICC indicated excellent reliability (≥0.90) with inter and intra‐observer error of <0.05 mm. The overall ICC was found to be high for assessing both translational and rotational changes as shown in Table 3. The mean absolute inter‐ and intra‐observer difference for translational and rotational changes was found to be less than 1 mm and 3°.

**TABLE 2:** Inter‐ and intra‐observer reliability of the registration validation part using intra‐class correlation coefficient (ICC) with mean absolute difference (MAD) and SD

	Part comparison registration	
	ICC	MAD ± SD (mm)
Intra‐observer	0.98	0.01 ± 0.01
Inter‐observer	0.90	0.03 ± 0.02

**TABLE 3:** Inter‐ and intra‐observer reliability using ICC with mean absolute difference (MAD) and SD

	Translational changes		Rotational changes	
	ICC	MAD ± SD (mm)	ICC	MAD ± SD (°)
Intra‐observer	0.99	0.32 ± 0.38	0.92	1.27 ± 1.12
Inter‐observer	0.98	0.24 ± 0.18	0.90	1.31 ± 1.09

Table 4 provides the mean translational and rotational changes of canine at 6 months post‐operatively in both cleft and non‐cleft side. The maximum translational displacement of the canines in both non‐cleft (1.5 ± 1.2 mm) and cleft side (1.4 ± 0.8 mm) was seen in an apical direction, and the minimum change was observed facially for the non‐cleft side (0.8 ± 0.4 mm) and mesially for the cleft side (0.1 ± 0.9 mm). For rotational changes the maximum change was observed for yaw with a clockwise rotation (−0.9 ± 3.6**°)** at non‐cleft side and (0.7 ± 4.5**°)** at cleft side with counter‐clockwise rotation. The minimum value at non‐cleft side was (−0.3 ± 2.1**°)** as pitch change with clockwise direction and in cleft side by (−0.3 ± 1.3**°)** as roll change with clockwise direction. The only significant difference noticed was for the translational changes in the facial/palatal direction.

**TABLE 4:** Mean and SD for the canine eruption in non‐cleft versus cleft side

	Translational changes (mm)			Rotational changes (°)			Volume (mm^3^)	
	Mesial/Distal	Palatal/Facial	Incisal	Pitch	Roll	Yaw	Preoperative	Registered postoperative
Non‐cleft side	−0.14 ± 0.49	0.75 ± 0.41	1.51 ± 1.15	−0.33 ± 2.12	0.56 ± 2.59	−0.92 ± 3.62	460.69 ± 95.08	446.22 ± 94.99
Mean ± SD								
Cleft side	0.14 ± 0.85	1.14 ± 0.70	1.35 ± 0.81	1.95 ± 3.32	−0.25 ± 1.27	0.69 ± 4.54	445.38 ± 118.47	437.58 ± 108.75
Mean ± SD								
Non‐cleft side versus cleft side (*p*‐value)	0.50	0.02*	1.0	0.08	0.73	0.92	0.32	0.43

* Statistically significant (*p* < 0.05); positive (+) sign in translational changes indicate mesial, facial and incisal direction; negative (−) sign in translational changes indicate distal, palatal and apical direction; positive (+) sign in rotational changes indicate counter‐clockwise rotation; negative (−) sign in rotational changes indicate clockwise rotation.

## 4 DISCUSSION

The evaluation of canine eruption in patients born with UCLP is essential for determining the optimal timing of the SABG and to avoid complications associated with canine impaction and eruption (Kaura et al., 2018). Various methodologies have been suggested for assessing the canine eruption in patients born with UCLP, however these have been carried out mainly in 2D and only one study has been reported in literature based on 3D cephalometry (Oberoi et al., 2010; Walker et al., 2005). Therefore, the current study was conducted utilizing a landmark‐free methodology for observing the post‐operative canine eruption in both cleft and non‐cleft side (Vellone et al., 2017).

In this study, we presented a novel registration‐based technique to assess the canine eruption by using six degrees of freedom i.e. translational (x, y, z dimensions) and rotational (pitch, roll, yaw) movements (Mulier et al., 2020). The results of the validation showed excellent reliability, as the ICC for both intra‐ and inter‐observer reliability ranged between 0.90 to 0.99. The overall error of the method was less than 0.4 mm for translational changes and less than 1.4° for rotational changes. This reduction in methodology error could have been associated with the use of a voxel‐based registration, validated segmentation tool and relying on semi to full automatic procedure, hence also lessening the human‐induced error (Almukhtar et al., 2014). Voxel‐based registration overcame the limitations of depending on landmarks or surfaces which have been considered to be more prone to human error (Almukhtar et al., 2014; Katkar et al., 2013). Moreover, the region of interest chosen for the registration step in this study was found to be reliable with an ICC ranging from 0.90 to 0.98 (observers' MAD <0.05 mm). These findings were in accordance with a study from Ruellas et al., also showing high reliability of the defined region of interest in growing patients (de Oliveira Ruellas et al., 2016). We also utilized three CBCT acquisition devices with different parameters for segmentation, which have been previously investigated by Shaheen et al. and found to be reliable for tooth segmentation without any significant difference (Shaheen et al., 2017).

For the normal canine eruption, Enrique et al. concluded that the transitional movement was seen in facial, mesial and apical direction (Fernández et al., 1998). In our study the canines on both cleft and non‐cleft side moved facially, mesially and incisally in all patients. A significant facial movement was observed in the cleft side compared to the non‐cleft region. This difference might have been related to the absence of buccal bone or resorption of SABG in the cleft side (Feichtinger et al., 2007). Except for the facial movement, no other significant difference was observed between the cleft and non‐cleft side related to the translational and rotational changes. For translational movement, our findings were comparable to that of Oberoi et al. (2010) in relation to the direction but not the amount of movement as they assessed the canine eruption 1 year post‐operatively. The mean rotational changes in non‐cleft side was less than that of the cleft side. However, cleft side showed the largest movement for yaw, followed by pitch and roll. At the same instance the reliability for rotational movement was found to be lower than translational movement, which could have been related to the systematic error (Junior et al., 2019). Unfortunately, no data was found in the literature enabling a comparison for rotational changes with our findings.

Several parameters can affect the upper canine's eruption, such as tooth agenesis or premature loss of deciduous teeth, which are highly prevalent factors in patients born with CLP. To avoid these parameters affecting our results, we tried to assure certain homogeneity during patient selection. In our sample, deciduous canines on the cleft side were either not present or extracted during the SABG surgery in all patients, and three patients had agenesis of the upper lateral incisor on the cleft side. Patients' individual dental and occlusal characteristics need to be taken into consideration when assessing the eruption of upper canines.

The sample size of this study could be considered a limitation for reaching a clinically‐oriented outcome. However, the sample size was considered adequate for validating the proposed methodology as it was based on similar validation studies with 3D assessment methodologies (Bianchi et al., 2010; Marchetti et al., 2011). Another limitation was the follow‐up time, which was limited to 6‐months postoperatively, yet timing was not the focus of our study. The present study was a validation study with a prime focus on accuracy assessment of following up the eruption process. This will now be further automated to create a clinically applicable, fast accurate tool. Nevertheless, to the best of our knowledge this is the first study to report on both translational and rotational changes of the canine eruption. Overall, our methodology proved to be clinically applicable, ensuring reliable and standardized data reporting of canine eruption. A larger sample size, longer follow‐up and timing are desirable for further validation of this methodology.

In conclusion, this study provided a reliable methodology for the 3D evaluation of canine eruption in patients born with UCLP. This methodology can now be applied in further studies including large trials with longer follow‐up time. The outcome of such studies would help to provide better insight related to the true 3D canine movements in both cleft and non‐cleft side. This would allow feedback on patient‐specific and surgically‐based outcome measures impacting the final orthodontic outcome.

## AUTHOR CONTRIBUTION

KA, ES, RJ. Study conduct: KA, ES. Data collection: KA, ES. Data analysis: KA, ES. Data interpretation: KA, ES, ME, TD, RJ. Drafting manuscript: KA, ES, SS, ML, CP, RJ. Approving final version of manuscript: all authors.

## CONFLICT OF INTEREST

The authors report no conflict of interest.

## ETHICS STATEMENT

Ethical approval was granted by the Local Ethical Committee (reference number: S58298).

## Data Availability

The data that support the findings of this study are available from the corresponding author upon reasonable request.
